# Sex Differences in Mouse Exploratory Behaviour to Fel d 1, a Cat ABP-Like Protein

**DOI:** 10.3390/ani11113149

**Published:** 2021-11-04

**Authors:** Carlos Grau, Cécile Bienboire-Frosini, Sana Arroub, Céline Lafont-Lecuelle, Julien Leclercq, Patrick Pageat

**Affiliations:** 1 Department of Chemical Ecology, Research Institute in Semiochemistry and Applied Ethology (IRSEA), Quartier Salignan, 84400 Apt, France; p.pageat@group-irsea.com; 2 Department of Behavioural and Physiological Mechanisms of Adaptation, Research Institute in Semiochemistry and Applied Ethology (IRSEA), Quartier Salignan, 84400 Apt, France; c.frosini@group-irsea.com; 3 Statistical Service Unit, Research and Education Directory Board Services, Research Institute in Semiochemistry and Applied Ethology (IRSEA), Quartier Salignan, 84400 Apt, France; s.arroub@group-irsea.com (S.A.); celine.lecuelle@sfr.fr (C.L.-L.); 4 Animal Research Unit, Research and Education Directory Board Services, Research Institute in Semiochemistry and Applied Ethology (IRSEA), Quartier Salignan, 84400 Apt, France; j.leclercq@group-irsea.com

**Keywords:** Fel d 1, predator-prey interactions, mouse behaviour, allelochemical, androgen-binding proteins, secretoglobin, sex differences, risk perception, chemical communication

## Abstract

**Simple Summary:**

Fel d 1 is a cat secreted protein, known as the main cat allergen, that is abundantly released and found in their habitat. Cats are one of the main predators of rodents and have been historically used to control rodent populations in human habitats. We assumed that laboratory mice, as a model of wild mice, would be able to detect and avoid this abundant cat molecule as a mechanism to increase chances of survival. In our study, we compared mice exploratory behaviours facing Fel d 1, a fox faeces molecule (TMT) as a positive control, and a negative control (purified water). We found that mice did not avoid Fel d 1 as we expected; however, male mice remained in the area with Fel d 1 longer than females. These results give interesting insights about how sexes can react differently to a predator stimulus and give support to the use of both sexes in behavioural studies, and more precisely in predator-prey interactions research

**Abstract:**

Fel d 1 is a cat protein abundantly released and found in their habitat and is closely related to mouse androgen-binding proteins (ABPs). We hypothesized that mice have developed chemical communication mechanisms to detect and avoid this protein. We tested purified natural Fel d 1, a fox faeces molecule (TMT) as a positive control, and a negative control (purified water) in three different mouse groups (*n* = 14 each) to evaluate exploratory behaviour and stress responses. The mice did not show clear avoidance or stress responses to Fel d 1. Our results demonstrated a sex-treatment interaction for Fel d 1, with males spending more time in the areas treated with Fel d 1 than in the untreated areas (*p* = 0.018). This sex-treated area interaction was also not observed for either the blank or TMT. These results suggest that Fel d 1 from domestic cats could be recognized differently by male and female mice. These sex differences could be linked to the sexual role of ABP proteins and the ABP-like characteristics of Fel d 1.

## 1. Introduction

The detection of predator cues is a valuable tool for survival, making this feature a criterion for selection throughout evolution. Predators and prey run a constant arms race that leads to increased fitness and survival [1], an evolutionary race that commensal species have perpetuated and accelerated in human habitats [2,3,4,5]. The domestic cat (*Felis catus*) is a major predator of the domestic mouse (*Mus musculus*) in human environments [6], which can favour the selection of prey individuals with phenotypes capable of detecting this predatory risk. More precisely, chemical communication plays a major role in predator detection [7], and protein chemical communication is well developed in mice [8]. 

Fel d 1, the major cat allergen, is a protein that is abundantly released by cats in its environment [9]. It is a member of the secretoglobin family and is characterized by small dimeric proteins capable of binding hydrophobic molecules [10]. It is mainly produced by the sebaceous glands from the facial area, skin, and anal sacs [11]. The main reservoir of Fel 1 is thus the fur of the cat, especially the cheek zones [12], as they are particularly rich in sebaceous glands [9]. In addition, it has been suggested that fur-derived odours could provide more valuable information to rodent prey than urine or faeces [13] since fur-derived odours tend to dissipate faster [14].

Papes et al. found that the cat protein Fel d 4 and the rat protein MUP13, which are members of the lipocalin family and are closely related to major urinary proteins (MUPs) from mice, induce avoidance and stress responses in laboratory mice [15]. They argued that duplicated gene coding for major urinary protein receptors from mice have evolved to detect similar MUP-like cat proteins, i.e., Fel d 4, which supposes an important evolutionary advantage with a relatively low biological cost.

With the same logic, we developed our research hypothesis. Androgen-binding proteins (ABPs) are proteins from the secretoglobin family that are implicated in intraspecific sexual communication in mice [16]. ABPs have a cat protein homologue from the same secretoglobin family, Fel d 1, with a related structure and function [17,18]. Therefore, we hypothesize that the high predatory pressure of cats in human environments could lead to adaptation (of duplicated genes from ABP receptors) for detecting the cat ABP-like protein Fel d 1 to increase fitness and survival.

In agreement with the anatomical origins and reservoirs of Fel d 1, May et al. [19] found that cat rubbing marks had an effect on Sprague-Dawley rats, including decreased feeding behaviour in partially food-deprived animals, increased hiding behaviour, and decreased exploratory behaviour. Cat rubbing behaviour includes facial and lateral body markings [20]. According to their results, the former authors suggested that this effect could be related to the cat protein Fel d 1.

Four assumptions can be made from the results of the previously described studies. First, cats’ living areas have a high concentration of Fel d 1, as has been widely demonstrated in the literature on allergology [11,21,22,23,24]. Second, cat rubbing marks showed a kairomone role in laboratory rodents [19]. Third, the anatomical areas involved in cat rubbing coincide with the main reservoir and production areas of Fel d 1 [12,25]. Fourth, Fel d 1 is closely related to ABPs, which are implied in sexual chemical communication [16,17]. Given these observations, it was hypothesized that Fel d 1 may have a kairomone role in mice and that both sexes could respond differently to this stimulus.

To test this hypothesis, we studied the effects of purified natural Fel d 1 on mouse exploratory behaviour, locomotor activity, and a stress-related parameter: the number of faecal boli. 

## 2. Materials and Methods

### 2.1. Animals

A total of 42 8–10-week-old C57BL/6JRj mice (21 males and 21 females, Janvier Labs, Genest-Saint-Isle, France) were kept at the facilities of the Research Institute in Semiochemistry and Applied Ethology (IRSEA) according to the requirements of French and European Law (2010/63/EU) and under the supervision of a veterinarian specialized in laboratory animals. The animals were not euthanized at the end of the experiments. The protocol and techniques described in this paper were approved by the IRSEA ethics committee (approval number AFCE20150501). 

The housing room was kept at a temperature of 22 ± 2 °C and 60 ± 20% humidity. A 12–12 h inverse (light: dark) light cycle regimen was used with the cycle beginning at 12:00 p.m. (lights off). Mice were habituated for two weeks to the inverse cycle before the tests. All procedures were conducted between 12:00 and 5:00 p.m., as the beginning of the dark cycle is one of the most active periods in mice [26].

The mice were housed in Eurostandard type IIIL cages (Tecniplast, Buguggiate, Italy) (369 mm × 156 mm × 132 mm), with a total surface area of 435 cm^2^. Animals were housed in single-sex groups per cage to minimize stress due to isolation, as mice are a social species; each cage housed three animals. Food was available ad libitum, supplied according to the 2014 global rodent diet (Envigo, Huntingdon, UK); the lignocel 3–4 bedding (Envigo, Huntingdon, UK) was changed weekly. As enrichment material, each cage was equipped with a red plastic tube along with craft paper and white paper as nesting material (Genobios, Laval, France), as mice prefer complex nests with more than one material [27].

### 2.2. Apparatus 

**Figure 1:**
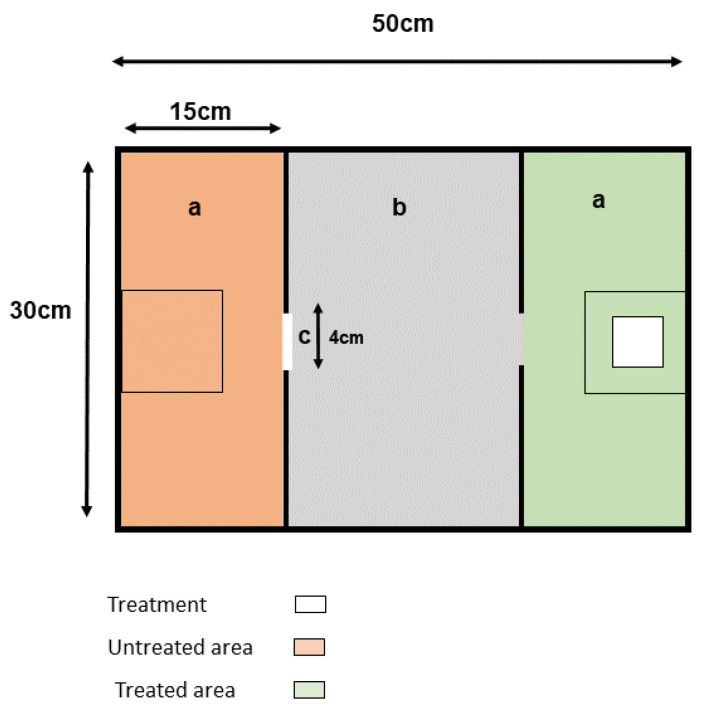
Top view schema of the device used for the behavioural tests. (**a**) Lateral areas where the treatment was randomly applied to one of the two sides; (**b**) central area where animals were released; (**c**) opening between the lateral and central areas.

Custom-built devices (Figure 1) were used as modified open fields to measure the house mouse’s exploratory behaviour, avoidance, and specific anti-predatory and fearful responses to predator stimuli as previously described [28,29,30]. Briefly, each device comprised a rectangular arena with a 4 mm thick, 50 × 30 cm glass base covered with a transparent plastic top. Squared glass was marked underneath with electric tape to distinguish two lateral and one central area in the arena. The treatment was applied to a piece of medical gauze and placed in one of the two lateral areas of the arena. The lateral areas were separated by a 1 mm thick opaque plastic PVC barrier measuring 24 × 30 cm, which was attached to the top of the arena. A small square (4.5 cm × 4.5 cm) was cut out in the centre to allow the test mice to move freely among the areas. Four identical devices were rotated for the replicates. 

The vertical plastic divisions reduced the passage of volatile compounds to adjacent areas and acted as a physical visual barrier.

### 2.3. Treatments and Treatment Application

The animals were naive to the tested stimuli, having had no previous contact with any of them. Each animal was used only once. The treatment was poured over a 4 × 4 cm piece of medical gauze, which was then placed over a square of glass (8 × 8 cm, 3 mm thick) to diminish contact with the arena and then placed in one of the two lateral sides of the arena. Treatments and treatment positions for each replicate were chosen according to a randomization procedure. 

#### 2.3.1. Blank

A piece of medical gauze without any treatment was used as the blank, as it was the carrier for the actual treatments as mentioned above. 

#### 2.3.2. Fel d 1 Protein

The cat secretoglobin Fel d 1 was provided as purified natural Fel d 1 (Indoor Biotechnologies, Cardiff, UK) after extraction from cat hair and purification by affinity chromatography. Cat hair used by Indoor Biotechnologies for Fel d 1 purification was provided by a cat veterinary clinic and was obtained from both sexes and several breeds. The total amount applied to the medical gauze was 7 µg diluted in 1 mL of ultrapure water, which is a realistic amount likely to be found in cat environments [31]. 

#### 2.3.3. 2,5-Dihydro-2,4,5-Trimethylthiazoline (TMT) 

2,5-Dihydro-2,4,5-trimethylthiazoline has been described as a volatile compound present in fox faeces [32] that elicits avoidance and fear responses in mice and rats [33,34]. Eight microliters of 90% pure solventless TMT (Srqbio, Sarasota, FL, USA) was used as the predator stimulus. This amount was based on previous literature where clear avoidance and fear responses were observed [15]. TMT was considered as the positive control for avoidance behaviour. 

### 2.4. Behavioural Test

All mice were habituated to the arena the day before the test for 10 min without any treatment. The tests were conducted in the experimental room between 12:00 p.m. and 5:00 p.m., with temperatures in the range of 21 ± 2 °C and 50 ± 20% humidity. The same operator handled the mice throughout all tests. The animals were transported from the holding cage to the arena using red PVC tubes to decrease stress from tail manipulation [35]. 

Animals were transported to a pre-test room at least 30 min before the experiments. They were then transported to the testing room, placed in the arena, and video recorded for 10 min. Every treatment group was composed of 14 mice (7 males and 7 females). 

A standard broad-spectrum lab disinfectant cleaner was used to clean the apparatus and its constituent elements between replicates. They were then cleaned with white paper towels dampened with water and dried with clean white paper towels. Ethanol was not used, as it elicits avoidance and modifies exploratory behaviour in laboratory mice [29]. Four identical arenas were rotated between replicates to dissipate possible volatile traces of cleaner product. The squares of glass where the treated gauze was applied underwent the same cleaning procedure but were used only once each day; at the end of the day, they were exposed to a pyrolysis treatment (480 °C for one hour) to eliminate residues. 

### 2.5. Measurements and Video Analysis 

Each replicate was video recorded with a video camera placed 1 metre over the arena (JVC HD Everio 1920 × 1080 full HD model GZ-HM446, Yokohama, Japan), located at a 90° viewing angle to the arena. This viewpoint allowed for a complete analysis of avoidance behaviour and locomotor activity. Video analysis was performed blindly by two independent observers (CG and JL). 

The avoidance behaviour was measured according to the total and average duration in all the areas of the device, the treated area, the untreated area, and the central area (Figure 1). Avoidance behaviour was considered when an animal significantly increased the total time it spent in the untreated area or decreased the total time spent in the treatment area. Similarly, regarding this main avoidance parameter, we measured the average time per passage in the treatment area and the untreated area, and we interpreted avoidance behaviour when animals decreased the average time per passage in the treatment area and/or increased the average time per passage in the untreated area. For a more detailed analysis of risk perception and exploratory behaviour, we measured the number of flight, freezing, risk assessment events [15], and the number of contacts with the stimulus. 

The total number of passages (defined as the total number of passages between areas) was counted as a locomotor activity related measure. The criterion to count an entry was when the 4 paws were in the zone. The number of faecal boli deposited in the arena was noted after each replicate as an independent parameter in the video analysis, and its average per group was used as a measure related to stress [36]. 

To ensure putative sensing of the purified Fel d 1 stimuli by mice, direct contact of the mouse nose with the medical gauze was verified during video analysis blindly for all treatments, as this behaviour has been described in the detection of other proteins in chemical communication in mice [8]. 

### 2.6. Statistical Tests

Data analysis was performed with SAS 9.4 (SAS Institute Inc; Cary, NC, USA). Before proceeding, the dataset reliability between the 2 independent observers was calculated. If normality was established, the Pearson correlation coefficient was used. Otherwise, the Spearman correlation coefficient was preferred. The acceptable inter-observer reliability was fixed at 0.9. 

Two different analyses were carried out: the first analysis compared the duration within the treated vs untreated area for each treatment and its interaction with sex. The second analysis compared the effects on avoidance behaviour, locomotor activity, contacts with stimulus, the number of faecal boli between different treatments, and their interactions with sex. 

In the first analysis, General Linear Model (GLM) procedure was used to compare the effects of sex and the area of the device (treated, untreated) and their interaction within each treatment (blank, Fel d 1, TMT). When significant differences were found, the Tukey test for multiple comparisons was performed using the Least-Squares (LS)means statement. 

In the second analysis, the effects of treatment and sex and their interaction were compared when conditions were verified using GLM procedure; otherwise, the Scheirer-Ray-Hare test was applied. When significant differences were found, the Tukey test for multiple comparisons was performed using the LSmeans statement. 

## 3. Results

The reliability between the observers who conducted the video analysis was greater than 0.9 for all the parameters, thus the average of the two observers was calculated.

**Figure 2:**
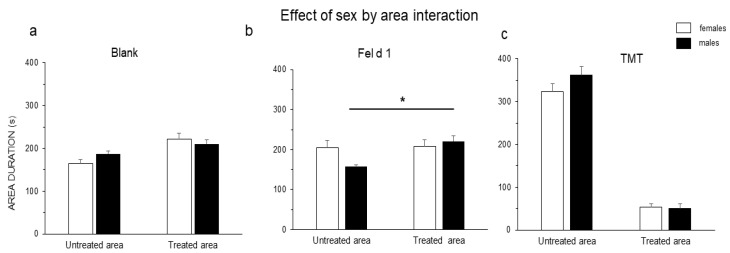
Durations in the treated vs. untreated areas for each treatment showing sex-by-area interactions (**a**–**c**). GLM procedure was used to evaluate the effects of the sex-area interactions, followed by a *Tukey* test and *LSmeans* comparisons. The results are shown as the mean ± SE. The significance threshold is fixed at 5%. *p* < 0.05 *.

A significant sex-by-area interaction was observed when treated vs. untreated areas were compared in Fel d 1 group, F(1,24) = 4.49, *p* = 0.045 (Figure 2b, GLM procedure). LS means showed that males spent significantly more time in the Fel d 1 treated area (220.7 ± 12.8 s) than in the untreated area (157.1 ± 4.7 s), t(24) = −3.22, *p* = 0.018; females did not show any significant difference between the treated (208.6 ± 15.2 s) and untreated areas (204.2 ± 18.9 s). This sex-by-area interaction was not observed for either the blank (Figure 2a) or TMT (Figure 2c). Considering only treatment, the mice spent significantly less time in the area treated with TMT (52.1 ± 5.9 s) than in the untreated area (343 ± 13.8 s), and this difference was highly significant, F(1,17) = 393.90, *p* < 0.001. The Sheirer-Ray-Hare test showed no difference in the central area between treatments, sex, and treatment-sex interaction (Figure S1a,b, Table S1).

**Figure 3:**
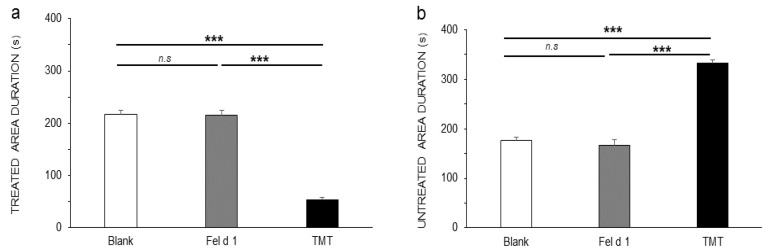
Comparison among treatments of the durations in (**a**) the treated area and (**b**) the untreated area. The analysis was carried out with the General Linear Model (GLM) procedure followed by a *Tukey* test and *Least Squares (LS) means* comparisons. The results are shown as the means ± SEs. The significance threshold is fixed at 5%. n.s, non-significant. *p* < 0.001 ***.

GLM procedure and Lsmeans comparisons showed that mice spent significantly less time in the area treated with TMT (52.1 ± 5.9 s) than with Fel d 1 (214.6 ± 9.7 s) or the blank (216.1 ± 7.8 s) F(2,11) = 132.5, *p* < 0.001 (Figure 3a) and conversely more time in the untreated area when the treated area contained TMT (343 ± 13.8 s) compared to Fel d 1 (180.6 ± 11.4 s) or the blank (175.6 ± 6.6 s), F(2,11) = 88.04, *p* < 0.001 (Figure 3b).

**Figure 4:**
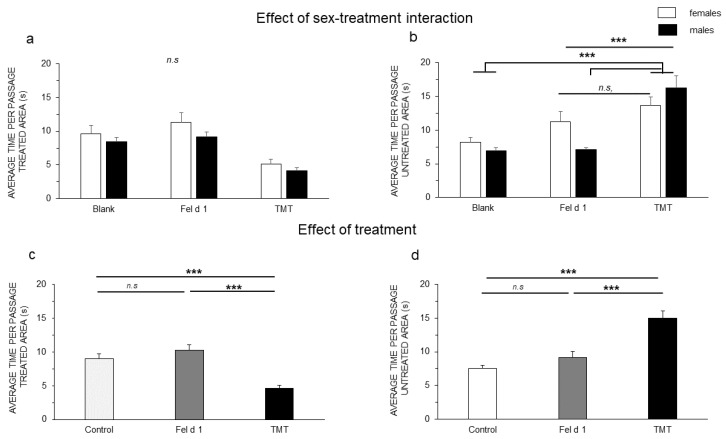
Average time per passage: (**a**) sex-treatment interaction effects in the treated; (**b**) untreated areas. (**c**) Effects of treatment in the treated and (**d**) untreated areas. Two-way ANOVA was used to evaluate the sex-treatment interaction effects, and LSmeans was used to test for multiple comparisons. The results are shown as the means ± SEs. The significance threshold is fixed at 5%. n.s, non-significant. *p* < 0.001 ***.

Results showed that the sex effect for the average time per passage in the treated area was not significant: F(1,40) = 3.89; *p* = 0.056, nor was the sex-treatment interaction: F(2,36) = 0.25; *p* = 0.78 (Figure 4a, GLM procedure). A significant interaction of sex and treatment was observed for the average time per passage in the untreated area (Figure 4b): F(2,36) = 4.46; *p* = 0.019; Lsmeans comparisons revealed that Fel d 1-treatment females (11.2 ±1.5 s) did not show a significant difference in the average time per passage in the untreated area compared to TMT females (13.6 ± 1.2 s) (*p* = 0.66), but all other combinations of sex and treatment showed significantly lower times compared to TMT (Figure 4b) (blank females (8.1 ± 0.7 s) vs. TMT females (13.6 ± 1.2 s), *p* = 0.018; blank females vs. TMT males (16.2 ± 1.7 s), *p*= 0.0002; Fel d 1 females (11.2 ±1.5 s) vs. TMT males, *p* = 0.036; Fel d 1 males (7 ± 0.2 s) vs. TMT females, *p* = 0.003; Fel d 1 males vs. TMT males, *p* < 0.001. A significant effect of treatment was observed for the average time per passage in the treated area, which was lower with TMT (4.6 ± 0.4 s) than with the blank (9.03 ± 0.6 s) and Fel d 1 (10.2 ± 0.8 s) (*p* < 0.001) (Figure 4c); inversely, the average time in the untreated area was greater for TMT (14.9 ± 1.1 s) than that in the blank (9.1 ± 0.9 s) and Fel d 1 (7.5 ± 0.4 s) untreated areas separately (Figure 4d).

**Figure 5:**
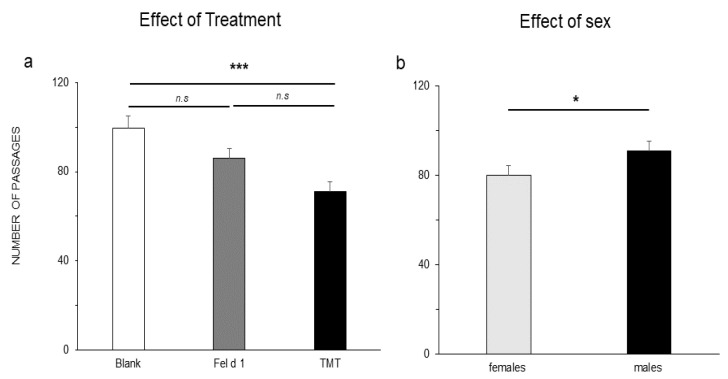
Number of passages between areas. (**a**) Effect of treatment; (**b**) effect of sex. The analysis was carried out with GLM procedure followed by a Tukey test and LSmeans comparisons. The results are shown as the means ± SEs. The significance threshold is fixed at 5%. n.s, non-significant. *p* < 0.001 ***, *p* < 0.05 *.

**Figure 6:**
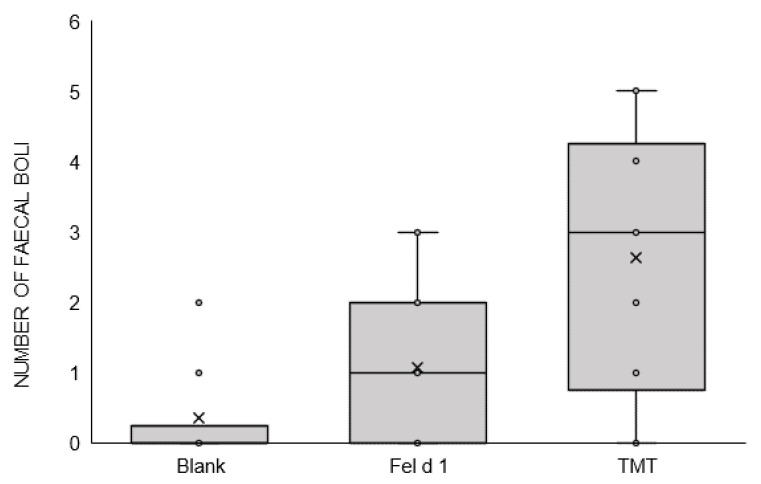
Number of faecal boli. Statistical analysis was performed with a *Scheirer-Ray-Hare* test (non-parametric conditions). The significance threshold is fixed at 5%.

A significant effect of treatment (F(2,39) = 9.43; *p* < 0.001) and sex (F(1,40) = 4.18; *p* = 0.048) but not of its interaction was observed for the number of passages by means of GLM procedure. Lsmeans did not show differences between Fel d 1 (85.9 ± 4.3 s) and the positive control TMT (71.2 ± 4.3 s) (*p* = 0.073) or the blank (99.39 ± 5.4 s) (*p* = 0.109). TMT showed a highly significant difference compared with the blank (Figure 5a) (*p* < 0.001). Females (80 ± 4.3) showed a significant lower number of passages than males (90.9 ± 4.4) (*p* = 0.048), independent of the treatment (Figure 5b). The number of faecal boli did not show any significant differences between treatments (Sheirer-Ray-Hare test) (*X*^2^ (2,42) = 5.08, *p* = 0.078) (Figure 6), 2.6 ± 0.49 (TMT), 1.07 ± 0.3 (Fel d 1), and 0.38 ± 0.2 (blank). Of note, the inter-individual variability was high for this parameter.

**Figure 7:**
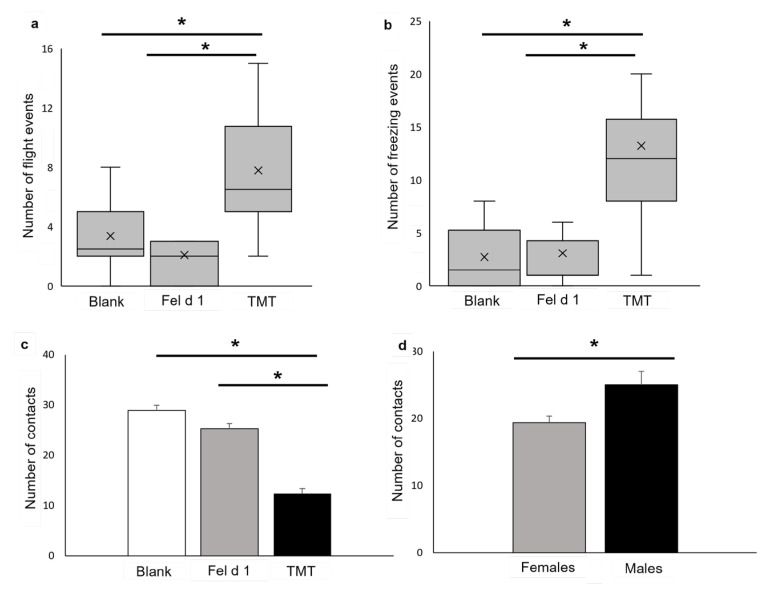
(**a**) Number of flight events; (**b**) number of freezing events. Number of contacts with the treatment: (**c**) effects of treatment; (**d**) effects of sex. Statistical analysis was performed Scheirer-Ray-Hare test (**a**,**b**) and with ANOVA and Lsmeans (**c**,**d**). The significance threshold is fixed at 5%. *p* < 0.05 *.

Flight behaviour (*X*^2^ (2,42) = 8.69, *p* = 0. 012) (Figure 7a) and freezing (*X*^2^ (2,42) = 9.81, *p* = 0.007) (Figure 7b) events were significantly higher for TMT than for Fel d 1 and the blank. However, no significant difference between sexes or treatments was found for the number of risk assessment behaviours (Figure S2, Table S2).

A significant effect of the treatment (Figure 7c) (F(2,39)= 42.28; *p* < 0.0001) and sex factors (Figure 7d) (F(1,40) = 13.55; *p* = 0.0008) was found for the number of contacts, but not for its interaction (sex-treatment). Contacts were significantly lower for TMT than for Fel d 1 and the blank (*p* < 0.0001), and males had a significantly higher number of contacts than females (*p* = 0.0008).

## 4. Discussion

Our results did not show an avoidance effect to Fel d 1; however, male mice were attracted to the protein, arising the putative sexual role of Fel d 1 and their ligands and its possible role in interspecific communication. 

Our study showed that natural Fel d 1, a cat secretoglobin produced in the secretory glands which role has been proposed in cat chemical communication [17,37,38] similar to that of the androgen-binding proteins in mice, induces different reactions in male and female mice. Males spent more time in the area where Fel d 1 was applied than in the untreated area; this effect was not observed in females. The average time per passage was not significantly different in the untreated area between females subjected to Fel d 1 and TMT (our positive control) treatments while it was lower than that of the TMT treatment for all the other combinations (blank/males and females, and Fel d 1/males). The hypothesis that Fel d 1 could have a kairomone effect was not clearly confirmed, as we did not observe avoidance or clear stress responses. On the other hand, TMT as a positive control, triggered clear and significant avoidance responses compared with the blank and Fel d 1 and decreased locomotor activity when compared with the blank and tended to elicit more faecal boli, thus validating our experimental design. However, the fact that a treatment-sex interaction was only observed with Fel d 1 suggests that it is indeed detected by mice males. Without further knowledge, this effect might lead to Fel d 1 being considered a putative chemical cue for mice [39]. 

The higher interest in males of the Fel d 1 stimulus may be linked to its close phylogeny and functional similarity to mouse androgen-binding proteins, which participate in sex discrimination in mice [16]. In addition, both proteins present different isoforms and an internal cavity that can contain volatile ligands (such as steroids) that are likely related to sexual communication [17,37,40]. Interspecies chemical communication with steroids has been described recently in mice [41], suggesting that testosterone attracted female mice and female brown rats equally, despite interspecies differences. In our study, Fel d 1 from both sexes of cats and different isoforms were used as olfactory stimuli. These isoforms can vary according to cat sex and behaviours as well as anatomical sites of production [25,42]. These results should therefore be framed as the response to Fel d 1 from a diverse cat population. 

Some studies have shown different reactions in males and females against predator stimuli [13], which have been justified by the greater reaction of females to predator stimuli. In our case, we observed that males had a higher number of passages than females (Figure 5a) that could shorten their average time per passage (Figure 4a). 

Regarding females’ behaviour, we did not observe a significant avoidance, but instead, attraction by the males to Fel d 1. In addition, we did not observe a significant interaction between sex and the other treatments, the blank or the predator control stimulus TMT; this interaction was specific to Fel d 1. Given these facts, it is difficult to justify our results from different behavioural traits between sexes; rather, it is more plausible that there exists different mechanisms of detection between the sexes. 

Purified Fel d 1 was significantly different from the positive control TMT in terms of predator stress-related behaviours (freezing and flight behaviours). In addition, the number of contacts with the stimuli was lower in TMT than with Fel d 1 and the blank, and mice showed avoidance only with the positive control. These results suggest than Fel d 1 was not perceived as a predator stimulus that could elicit a fear response. Interestingly, Fel d 1 results were not different from TMT in unspecific stress related parameters (number of faecal boli and number of passages) (Figure 5 and Figure 6); furthermore, these behaviours showed intermediate values between those elicited by TMT as a positive control and those elicited by the blank. TMT did not show a significant difference compared to Fel d 1 in terms of locomotor activity while this parameter was significantly lower for TMT than for the blank. For the number of faecal boli, we observed the same patterns in the results: a greater response to TMT followed by Fel d 1 and the blank, with a statistical trend but not a significant difference, likely due to the high variability in this parameter. We cannot discard that mice detected Fel d 1 as a signal of danger and showed a mild stress response but not avoidance, as it has been showed in other studies for predator stimuli [34,43,44]. It could be possible that higher amounts of Fel d 1 than those tested in this study could elicit stronger reactions, by means of a higher predator-pressure perception. Further research could explore the response to both proteins combined Fel d 1 and Fel d 4 as they are found together in cats’ environment [45] and could give mice more complete information about their predators. 

May et al. [19] found an effect of cheek rubbing marks from domestic cats in rats: feeding behaviour was reduced compared to controls and avoidance behaviours increased. Fel d 1 is particularly present in the facial area of cats due to the high density of sebaceous glands [9,12]. However, the authors did not identify the molecular content of the rubbing marks during their study and only studied those from a single cat. Fel d 1 production can vary greatly between subjects [31,42]; therefore, it is not possible to confirm the presence of this protein in these experiments. Consequently, the anti-predatory response observed by May et al. [19] could have been elicited by compounds other than Fel d 1. In addition, we should notice that perception of these molecules could differ between rats and mice due to its ancient phylogenetic divergence [46].

The fact that male mice preferred to spend more time in areas where Fel d 1 was present could be considered a disadvantage as in nature they would be more exposed to predation in cat areas. This could be partially due to boldness or a different perception of risk-benefits balance, as we observe in Figure 7 with a higher number of contacts with the stimuli. Use of shared or similar compounds (mimicry) in chemical ecology in benefits of the predator is better known in invertebrate species [47] A similar approach of counterespionage has been described recently in mice; male mice and rats were attracted by progesterone and estradiol and females from both species were attracted by testosterone [41,48]. We should consider a possible effect of inner ligands of Fel d 1 related to sexual communication (steroids) in male mice attraction [37], added to the phylogenetic and structural proximity between ABPs and Fel d 1. 

Our results highlighted the importance of using both sexes in interspecific chemical communication studies in mammals, as many studies have historically included only one sex to decrease the hypothetical variability linked to hormonal estrus variation in females [5,6]; however, this concept limits the external validity and generalization of such studies to a high degree. Dey et al. found that female mice responded differently to male MUPs depending on the estrus cycle stage [49,50], showing attraction during estrus and indifference during diestrus. However, no difference during the estrus cycle has been observed against the cat protein Fel d 4 as a predator stimulus, and in a previous study, no difference was found between male and female mice reactions to Fel d 4 [15]. Interestingly, we observed different reactions between sexes to cat protein Fel d 1. 

## 5. Conclusions

To our knowledge, this is the first study that suggests an allelochemical role for Fel d 1, a member of the secretoglobin family, and that has reported different sex responses to proteins in interspecific communications in mice. Sex is an outstanding factor in intraspecific chemical ecology; however, its role in interspecific communication remains to be determined. 

## Data Availability

All relevant data is shown in the main body of the manuscript.

## Supplementary Materials

The following are available online at https://www.mdpi.com/article/10.3390/ani11113149/s1, Figure S1. Duration in the central area per sex and treatment (a) and per treatment (b). Sheirer-Ray-Hare test. Significant threshold was fixed at 0.05. Table S1. Statistics for central area. Figure S2. Number of risk assessment behaviours during the tests. Sheirer-Ray-Hare test. Significant threshold was fixed at 0.5. Table S2. Statistics for risk assessment behaviour.
