# Supplementary material for: Sex Differences in Mouse Exploratory Behaviour to Fel d 1, a Cat ABP-Like Protein
Source: Animals (Basel). 2021 Nov 4;11(11):3149. doi: 10.3390/ani11113149 (PMC8614430; doi:10.3390/ani11113149)
Supplement: Supplementary file 1 [file animals-11-03149-s001.zip › for proof-supplementary -animals-1410187.pdf]

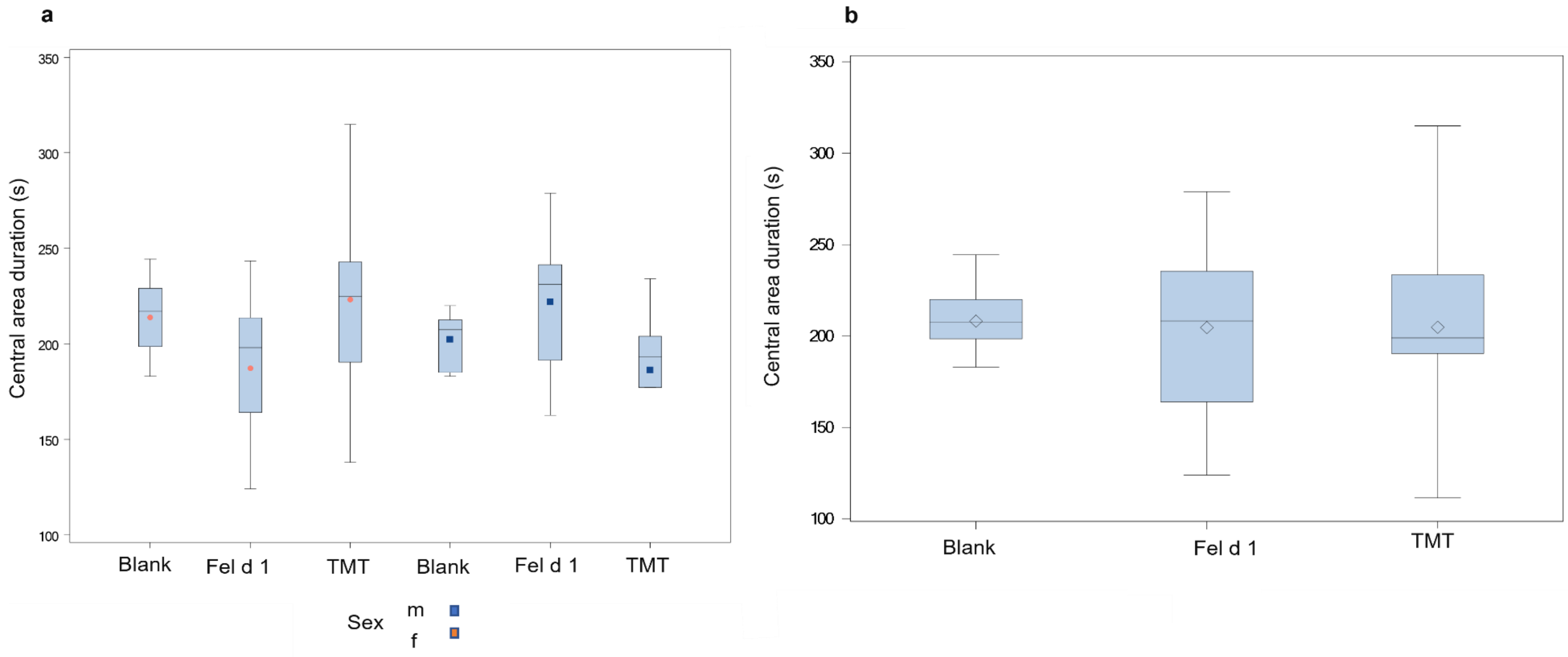

Figure S1. Duration in the central area per sex and treatment (a) and per treatment (b). Sheirer-Ray-Hare test. Significant threshold was fixed at 0.05.

Table S1. Statistics for central area

Variable	Mean-squared	X^2	*p*
Treatment	5,786	0.038	0.980
Sex	46.095	0.306	0.579
Treatment × Sex	477.310	3.171	0.204

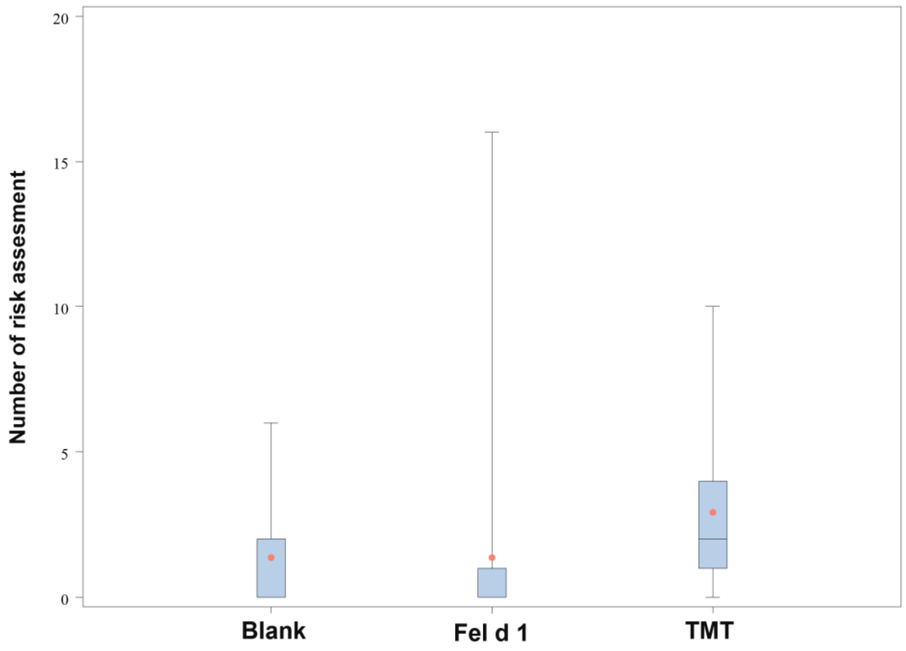

Figure S2. Number of risk assessment behaviours during the tests. Sheirer-Ray-Hare test. Significant threshold was fixed at 0,5.

Table S2. Statistics for risk assessment behaviour

Variable	Mean-squared	X^2	*p*
Treatment	546.232	3.629	0.162
Sex	17.357	0.115	0.734
Treatment × Sex	149.589	0.993	0.608
